# Exploring the motivations, challenges, and barriers for implementing evidence-based veterinary medicine (EBVM) in general practice

**DOI:** 10.18849/ve.v8i1.602

**Published:** 2023-03-16

**Authors:** Laura Haddock+, Sarah Baillie+, Ellie Sellers+, Sheena Warman+

**Keywords:** EVIDENCE-BASED VETERINARY MEDICINE, VETERINARY PRACTITIONERS, ONLINE LEARNING RESOURCE, EBVM

## Abstract

An evidence-based veterinary medicine (EBVM) training resource, ‘EBVM Learning’, was created in 2015 and updated in 2019. Following feedback from users, it was decided that a more concise practitioner-focused version was needed.

Seven online focus groups, with a total of 22 veterinary practitioners, explored the motivations of practitioners to engage with EBVM. They considered the challenges and barriers to implementing EBVM in practice, and specific supportive measures they felt would increase adoption of EBVM in practice. Participants identified time, support from colleagues and management, and accessing and appraising evidence as being the most important challenges and barriers to the use of EBVM in general practice. However, the value of EBVM was appreciated, and there was an appetite amongst the participants to utilise more EBVM to inform their clinical decision-making.

At a workshop attended by experts in EBVM, educators and practitioners, the results of the focus groups were presented and discussed to inform the development of a new online training resource.

This study has been used to produce ‘EBVM for Practitioners’, to attempt to reduce some of the barriers and challenges faced by practitioners and support them in increasing their use of EBVM. Further work by the leaders in the veterinary profession will be needed to expand and improve the quality of the evidence base on which EBVM relies, and to ensure practitioners have the skills, access, and motivation to utilise it.

## Introduction

There is a widely held societal expectation that diagnostic and therapeutic decisions made by practitioners are based on evaluation of high-quality scientific literature, a practice known as Evidence-based Veterinary Medicine (EBVM) (Shaw, 2001; Heneghan et al., 2017; and Janicke et al., 2020). EBVM has been defined as the conscientious, explicit, and judicious use of current best evidence to make the best possible decision about a patient (Sackett et al., 1996; and Dean & Heneghan, 2019). Practitioners go beyond this to contextualise their decisions and actions within a broader professional framework, considering the individual circumstances of the clinical scenario, patient, and owner (Centre for Evidence-based Veterinary Medicine, n.d.).

EBVM is becoming increasingly important to veterinary practitioners and is now included within the core competencies defined by some of the accrediting bodies of veterinary education (e.g., American Veterinary Medical Association (AVMA); Royal College of Veterinary Surgeons (RCVS); and European Association of Establishments for Veterinary Education (EAEVE)). Incorporating EBVM skills within clinical decision-making enables practitioners to be more efficient in their use of evidence-based practice (Sackett, 1997; Doig & Simpson, 2003; and Huntley et al., 2017) and helps to build trust with their clients (Hauser & Jackson, 2017). It is important that practitioners can make judicious use of EBVM as their clients have increasing access to online information (Steele et al., 2013; and Gibbons et al., 2021). This increased client expectation and knowledge can create a major source of stress for practitioners (Gardner & Hini, 2006).

It is widely recognised that clinicians face several barriers to using EBVM. The most significant of these are time, access to quality evidence, and making evidence relevant and applicable to the clinical setting (Sackett, 1997; Doig & Simpson, 2003; Turner & Royle, 2015; Huntley et al., 2016; Hauser & Jackson, 2017; Gibbons et al., 2021; and Sellers et al., 2021). Practitioners also need the necessary skills to practise EBVM; effective literature searching, critical appraisal, and the ability to apply the evidence found within the clinical context (Vandeweerd et al., 2012; and Huntley et al., 2017). These skills are essential to both identify high-quality evidence and ensure evidence gathering is efficient (Vanderweerd et al., 2012).

Veterinary practitioners are confronted with a lot of readily available online information sources (Nielsen et al., 2015; and Gibbons et al., 2021), and a study in 2012 revealed there were 1139 journals with a significant amount of veterinary content (Grindlay et al., 2012). However, research suggests that veterinary practitioners preferred to use online resources such as the Veterinary Information Network (VIN, 1991.) and the International Veterinary Information Service (IVIS, 1998.), or to ask a colleague for advice (Vandeweerd et al., 2012; Nielsen et al., 2015; and Huntley et al., 2016).

A need for improved access to evidence is frequently cited in the veterinary literature (Wales, 2000; Vandeweerd et al., 2012; Hauser & Jackson, 2017; and Sellers et al., 2021). A decade ago, it was reported that there was significant underdevelopment in the veterinary field across all elements of the information infrastructure that supported EBVM when compared to that of human medicine (Toews, 2011). A further report makes a strong case for much-needed funding for research to grow the evidence base (Lanyon, 2014) and, more recently, to increase the number and availability of evidence summaries in practice (Brennan et al., 2020).

In response, and following the lead of human medicine, there have been considerable developments in EBVM. Evidence syntheses (e.g. systematic reviews and evidence summaries)rovide an efficient means of accessing succinct syntheses of clinically relevant information in a standardised format (Dean et al., 2015; and Brennan et al., 2020). An online database listing veterinary systematic reviews has been developed (VetSRev, 2020) and there are increasing numbers of freely accessible online evidence summaries (e.g., BestBETS for Vets; Knowledge Summaries; and Clinical Evidence in Equine Practice).

Other initiatives include freely available online databases to search for research papers (e.g., PubMed), open-access journals (e.g., *Veterinary Record Open*, and *Veterinary Evidence*) and evidence-based practice guidelines (e.g., Fletcher et al., 2012; Sparkes et al., 2015; and Carney et al., 2016). Veterinary practitioners can subscribe to libraries of resources focused on veterinary science and animal health (e.g., RCVS Knowledge Library). Ventures such as SAVSNET and VeNom Coding utilise practice-generated data to answer specific clinical questions and act as a form of disease surveillance (Kerbyson, 2015).

EBVM is gaining interest across the profession, with quality improvement being incorporated as an expected part of regulated practice standards in the UK (RCVS Practice Standards Scheme, 2022). There has been an increase in training in EBVM skills in undergraduate veterinary curricula (Arlt et al., 2012; Steele et al., 2013; Cake et al., 2016; Shurtz et al., 2016; Dean et al., 2017; Sellers et al., 2021; Gibbons et al., 2021; and Batt-Williams & Lumbis, 2022), along with the creation of freely available online resources to assist educators e.g. how to include EBVM and research skills in veterinary training (Janicke et al., 2020). Reports suggest that there are an increasing number of graduates with EBVM skills entering the workforce (Hauser & Jackson, 2017; and Huntley et al., 2017). However, the impact of historically little formal training has led to varying levels of awareness and skills within the profession (Sellers et al., 2021).

In a recent international survey, 3660/5255 (69%) of veterinarians reported a desire to hear more about EBVM (Huntley et al., 2017). In the same research, it was noted that continuing education plays an important role in bringing EBVM skills to those who did not receive formal teaching on EBVM in their undergraduate curriculum. There are postgraduate training opportunities available to develop EBVM skills e.g., the freely available online course ‘EBVM Learning’, as well as via formal courses, but these require time investment from busy practitioners.

In a previous study, feedback was gathered on the online resource ‘EBVM Learning’ from those using it in academia and practice (Sellers et al., 2021). The information was used to review and update the resource to ensure it remained relevant and valuable for the range of learners engaged in EBVM. The project also identified challenges, real and perceived, encountered by practitioners in their attempts to embed EBVM into clinical practice e.g., time, access to evidence, and confidence in their own EBVM skills. As a result, it was proposed that a different training resource was needed for practitioners, one that did not cover the same depth and scope as ‘EBVM Learning’. It could provide training in the basic principles of EBVM in a manageable concise format, while linking to the ‘EBVM Learning’ resource for further detail.

Practitioners need a concise and focused EBVM tool to support upskillingthis needs to be internationally and freely available to stimulate further interest and engagement in EBVM (Vandeweerd et al., 2012; and Huntley et al., 2017). The current study aimed to explore the themes identified previously by practitioners in more depth and use the results to inform the development of ‘EBVM for Practitioners’: a concise, practitioner-focused version of ‘EBVM Learning’.

## Methods

### Focus groups

A series of focus groups (Krueger & Casey, 2014) was used to discuss three topic areas: motivations of practitioners to engage with EBVM; the challenges and barriers to implementing EBVM in practice; and specific needs to support increased adoption of EBVM in practice in the future. The focus group questions are in Appendix 1. The study received approval from the Faculty of Health Sciences Research Ethics Committee, University of Bristol (Reference 99803).

Participants were recruited using a convenience sampling strategy by approaching veterinary practitioners (veterinary surgeons and veterinary nurses) working in general practice from within the project team’s networks. Efforts were made to include those working with different species, from both independent and corporate practices, and from a range of geographical locations in the UK.

A recruitment email and information sheet were sent to potential participants and those agreeing to attend a focus group signed a consent form.

Due to the Covid-19 pandemic, the focus groups were undertaken online (using the Skype video conferencing platform) and all were facilitated by the lead researcher (author LH, an experienced primary care veterinary practitioner). Each focus group was restricted to a maximum of four participants to allow for meaningful contributions in the virtual environment. Seven focus groups were undertaken with a total of 22 participants (Table 1).

**Table 1:** Summary information for the focus group participants

Focus Group Demographics		
Age		
Mean	33.6 years	
Range	24–56 years	
Graduated		
2010–2019	14	63.6%
2000–2019	5	22.7%
1990–1999	2	9.1%
1980–1989	1	4.6%
Before 1990	0	0%
Mean years graduated:	9.5 years	
Gender		
Male	6	27.3%
Female	16	72.7%
Geographical location		
England	18	81.8%
Scotland	1	4.6%
Wales	3	13.6%
N. Ireland	0	0%
Practice ownership type		
Independent	13	59.1%
Corporate	7	31.8%
Charity	2	9.1%
Practice type		
Small animal	15	68.2%
Equine	4	12.8%
Farm animal	3	13.6%
Job role		
Veterinary surgeon	20	90.9%
Veterinary nurse	2	9.1%

The focus groups were audio-recorded, and then transcribed by a university-approved commercial service (University Transcriptions). Each participant was assigned a number (1–22) and their contributions were labelled with their number prior to analysis to anonymise them.

### Analysis

The transcripts were read twice by LH. One transcript was then selected and analysed independently by two researchers (LH and SW) to identify themes and sub-themes within each topic area (1. Motivations; 2. Challenges and barriers; and 3. Future needs). The data were hand coded using a mixture of deductive and inductive thematic analysis (Braun & Clarke, 2006), identifying codes throughout the transcript where relevant to any of the main topic areas, then grouping these codes into themes and sub-themes. The themes and sub-themes within each topic area were discussed between the two researchers to reach a consensus for an initial thematic framework. This was followed by full coding of each transcript by LH, during which a few additional themes and sub-themes emerged; representative quotes were allocated to each.

### Researcher Positioning

Researcher positioning is an important consideration in qualitative research. The two authors responsible for data collection and analysis have backgrounds as an experienced primary care clinician and secondary school teacher (LH) and an experienced referral clinician, educator and qualitative educational researcher (SW). This ‘insider-outsider’ positioning (Dwyer & Buckle, 2009) is acknowledged as having potential to influence interpretation of the data; the authors took a reflexive approach to data collection and analysis, using questioning and discussion to critically reflect on the potential impact of researcher positioning and presuppositions on the research findings.

### Workshop

A workshop was hosted by RCVS Knowledge via Zoom where the focus group results were presented. Attendees included experts in EBVM, educators and practitioners, some of whom had been focus group participants. Breakout rooms were used for small groups to discuss the focus group results and the design of a new online resource specifically to support practitioners in their use of EBVM. The groups were to make suggestions about the structure, content, and key differences between the new resource and ‘EBVM Learning’.

### Results

The final thematic framework (Table 2), was developed and agreed through iterative discussion by LH and SW.

**Table 2:** Final thematic framework

Main Themes	Subthemes
1. Motivations: Understand what motivates practitioners to engage with EBVM	Understanding the concept of EBVM
	Previous experience with EBVM
	Current awareness and use of EBVM
	Perceived benefits of EBVM
2. Challenges and barriers: Gain a greater understanding of the challenges implementing EBVM in practice	Time
	Support
	Accessing and evaluating evidence
3. Future needs: Identify specific needs to support future adoption of EBVM in practice	Requirements of an online learning resource
	Other ways to remove barriers / challenges

### 1. Motivations: Understand what motivates practitioners to engage with EBVM

### Understanding of the concept of EBVM

Participants across all focus groups agreed that EBVM supported continuous improvement in clinical practice, leading to better outcomes for clients and patients. Each participant was assigned a number (1–22) and their contributions were labelled with their number prior to analysis to anonymise them (seen in brackets after each of the focus group quotes below).

‘[EBVM is] best practice, research based, where you’re looking at outcomes and providing good clinical judgements and probably implementing treatment regimes and preventative medicine based on good evidence’. (1)

EBVM was seen as something that was often practised by those keen to keep up to date and could be used to provide evidence to support positive change. Many participants felt that EBVM was relevant to those working in general practice, although some felt it needed to be used more.

‘I wish it was implemented more […] I think it’s really important to do and I think it just gets overlooked sometimes’. (9)

### Previous experience of EBVM

Participants who had graduated recently, and those currently involved in teaching or mentoring, were aware that EBVM is now embedded in most undergraduate curricula. However, those who graduated a while ago reported receiving little teaching on EBVM as students.

‘I don’t think [EBVM] was a term when I was studying’. (16)

Most practitioners who had undertaken postgraduate study felt that it included some exposure to EBVM, but others felt that it was not a priority in the programme of study they had undertaken.

‘It did come up slightly [in my Certificate], but I don’t think there was a full lot of learning on that’. (15)

It was suggested by several participants that those who studied EBVM at university / college were more likely to want to take part in EBVM-related activities in practice.

‘[…] its use increases as graduates come through the system, because it’s incorporated into the syllabus of the veterinary courses, and the graduates arrive expecting protocols [...]’. (1)

### Current awareness and use of EBVM

Participants described the sources of evidence that they commonly use; these included discussion with colleagues, or sharing evidence within group settings e.g., journal clubs / rounds. Specialist opinion was also mentioned frequently as being highly regarded, with the perception being that such opinions would be backed by evidence but also grounded in clinical experience.

‘[Specialist opinion] not only comes with that kind of evidence from the papers but it also comes with that specialist experience as well, which gives you the confidence to go for it […]’. (5)

Several participants mentioned the value of discussing cases with new graduates and students, along with reflecting on their own previous relevant experiences. Other popular sources of evidence were textbooks, Continuing Professional Development (CPD) events, or articles published in veterinary magazines. A few participants mentioned using journals as a source of evidence, either by searching journals for papers on specific subjects, or by browsing a particular journal to keep abreast of developments.

‘I do read journals […] I’ll choose out the bits that are of interest to me and my working life’. (20)

Searching for research papers by using an online search engine was mentioned by only two participants and accessing published research via a database was mentioned by only one. Several participants mentioned that they were aware of the existence of evidence summaries in a variety of formats.

The participants widely acknowledged that the quality of evidence from different sources is variable, and the more convenient sources (colleagues, textbooks) could be less reliable.

‘Looking up in the books as well, but I think the problem with the books is that sometimes they are a few years old and there isn’t up to date thinking in them’. (22)

Almost all participants were able to list some specific EBVM activities that they undertook within their practice. There was a clear appetite for the use of evidence-based protocols and clinical guidelines. To maximise the confidence practitioners had in their in-house protocols / guidelines they wanted the method of evidence-gathering to be transparent, and for there to be regular reviews of the evidence.

‘I think probably some of our SOPs [Standard Operating Procedures], we’ve got a lot of SOPs, and I think some of them were, once upon a time [evidence-based], whether they still are now […]'. (19)

Consensus guidelines, such as recommendations on appropriate antibiotic use (PROTECT [BSAVA & SAMSoc, 2018]), and mortality and morbidity rounds were mentioned as ways to use EBVM to improve future outcomes.

‘[…] with the mortality and morbidity rounds, that’s really addressing things that have come up. And learning and then developing protocols because of those’. (12)      

A commonly mentioned activity was group discussion within the clinical team about cases or research papers (variably termed journal clubs, case discussions, clinical clubs, and clinical veterinary meetings). These group knowledge-sharing activities were seen as an expected part of clinical practice, and most participants felt they were useful and rewarding.

‘We do journal clubs and case discussions with senior clinicians that know a lot more than I do […] I try and get everybody involved in – we have it every week without fail […] It is a planned event, as part of the weekly schedule[…]’. (4)

Although audits are now an integral part of practice standards, most of the focus group participants were not actively involved in the audit process within their practice. A few mentioned that more of their team were starting to be involved in the audit process, and several felt that it would be used more in the future.

‘[…] all our senior vets and senior [veterinary] nurses have been split into groups and we’ve actually got kind of a clinical audit team now. So, I think […] we will start to use it more in practice’. (21)

Only a couple of practitioners mentioned using EBVM to make decisions proactively for individual cases.

### Perceived benefits of EBVM

Overall practitioners felt that there were many benefits to increasing the use of EBVM for individuals, practices and the profession. The perceived benefits included supporting clinical decision-making, improving job satisfaction, reducing stress, improving efficiency, increasing confidence, better outcomes for patients, and improved animal health and welfare.

‘[…] it does feel very good when you get it right and evidence-based vet medicine will help you get that goal’. (18)

Some of the practitioners also felt that EBVM could lead to a more cost-effective approach for clients, for example; by leading to the correct treatment more quickly, thus improving client-practitioner relationships.

‘[…] you’re doing the best that you can do for the patient and offering the best and most economical outcome for the owner as well’. (11)

Within farm practice, this was seen as a significant commercial benefit.

‘[…] if you can prove there’s a cost benefit as well, I think that definitely helps’. (2)

Many of the participants felt that EBVM led to a more consistent approach across the veterinary team, improving the client’s confidence and leading to a more satisfied and cohesive veterinary team.

‘[…] if they go and see two different vets in the same practice within a couple of days and each vet is telling them a different thing, that can be quite confusing, and I don’t think they have enough trust in us then […]’. (22)

Many of the benefits described within this theme could increase practice profits, along with client retention.

‘Client satisfaction will drive increased business’. (8)

Several practitioners felt that EBVM would enhance the reputation of the veterinary profession by improving the quality of veterinary care, and by stimulating further research.

‘[…] if we want to ask the questions that we want the answers to, that would definitely help to drive research in the right direction […] and it stimulates more studies to be done’. (20)

Some benefits of EBVM were considered to be broader than just the veterinary profession, in particular the responsible use of antimicrobials.

‘[…] if you’re using things like rational use of antibiotics, you’re protecting the human population as well as other animals […]’. (15)

### 2. Challenges and barriers: Gain a greater understanding of the challenges implementing EBVM in practice

### Time

The most significant challenge that practitioners identified was the time required to engage in EBVM. The focus group participants often replied simply with the word ‘Time’ as a response to queries about challenges and barriers, with many participants feeling no need to provide any further explanation. The most commonly cited time-consuming activities were finding evidence, reading literature, discussing CPD, and generating protocols. Some alluded to a sense of the whole process being overwhelming.

‘It is time, I think. That is the main restriction. It’s having time to discuss CPD that’s been learned, to read what other people have written and all the rest of it’. (11)

‘Time, without a doubt […] I don’t have time to go reading journal after journal after journal after journal, to make one decision on whether I should do a flank cat spay or a midline cat – those sorts of things, it just takes too long’. (15)

### Support

A lack of support presented some challenges, whether related to being allowed paid time to learn more about EBVM or meeting resistance from colleagues who were reluctant to change established practices.

‘I think sometimes people are set in their ways […] people that have been in practice for longer […] they set the protocol, so, it’s harder for you then to make changes’. (12)

Several participants reported that some colleagues did not like protocols or clinical guidelines, as they felt they restricted clinical freedom. Some reported being disheartened after undertaking the initial stages of EBVM, when reluctant colleagues prevented changes being applied in practice. Although a few were more optimistic that some activities, specifically audits and journal clubs, would be supported and well received.

‘I think if it was presented in the right way and for the right thing, they might consider it’. (19)

### Accessing and evaluating evidence

Gaining access to evidence could be challenging, often requiring a journal subscription, and sometimes the evidence required does not yet exist.

‘[…] but also access is a major problem, because access costs money’. (20)

Many also mentioned that they did not know where to look, or how to gain access quickly and easily.

‘And I think it is quite difficult to actually look up stuff. So, it can be challenging to find the articles, to find the knowledge about certain topics’. (22)

Whilst inclusion of specific training on the EBVM process varied across the spectrum of postgraduate training the practitioners had undertaken, most who had completed formal postgraduate study had gained additional experience of acquiring and appraising evidence, and so felt more comfortable with these aspects of the EBVM cycle as a result.

‘My knowledge of appraising evidence is based on what I’ve been taught as part of my internship and since working in a referral hospital’. (4)

Frustration was expressed that some of the evidence available in veterinary medicine was perceived to be of low quality; examples included small, poorly conducted, or biased studies, or articles written based on opinion rather than evidence. Additionally, sometimes the evidence was not directly applicable to their own clinical practice.

‘[…] a lot of the case selection of published papers are through referral hospitals and second opinions, so it’s quite hard to find a lot of first opinion data […]’. (15)

‘And particularly in more extensive systems beef and sheep, the amount of peer reviewed papers available to you, to answer a specific question are actually quite few’. (1)

Another concern related to assessing the impact of evidence when applied in practice, as without feedback mechanisms it was not clear if the associated changes had had a positive impact.

‘I also feel there’s less auditing done in veterinary practice […] so no-one knows really what we’re doing and what the outcome is in general practice. You might have people researching things, but there’s not that much that we feedback as to what worked and what didn’t work’. (12)   

It was noted that one of the challenges in veterinary medicine (compared to human medicine) was an insufficient quantity of evidence to draw meaningful conclusions.

‘[…] often the outcome is that, actually, the evidence that we’ve got doesn’t support either way […]’. (14)

Once evidence had been obtained, participants varied in how confident they felt in their ability to understand or appraise it. Consequently, some felt that their lack of skills and previous training in these areas discouraged them from using EBVM. Several mentioned relying on the process of peer-review as reassurance that the evidence was of sufficient quality.

### 3. Future needs: Identify specific needs to support future adoption of EBVM in practice

The final area explored in the focus groups was ways in which future developments, and specifically the planned online learning resource, could assist practitioners in incorporating more EBVM in general practice.

### Requirements of an online learning resource

Participants were asked to review the existing online ‘EBVM Learning’ course prior to the focus groups. Their feedback indicated that the resource was very good, but it was too detailed for their needs.

‘[…] I read through the first section, but I didn’t go through the whole thing. I think a shorter one probably for me […] I feel like the five A’s thing is really good, but it is just a lot […]’. (2)

They were then asked to consider a new practitioner-focused version. Nearly everyone mentioned that the resource needed to be concise, as the main barrier to EBVM use in practice is time.

‘I think it needs to be short and sweet’. (18)

The type of content requested varied with the previous experience and current skill level of the participants. For those who had not encountered EBVM as undergraduate or postgraduate students, training in how to acquire and appraise evidence were frequently mentioned. In terms of acquiring evidence, suggestions were to focus on the most relevant sources, preferably with free or affordable access. Overall, it was suggested that one way to encourage completion of all five stages of EBVM was to provide a proforma with clinical relevance for each type of EBVM activity e.g., creating a Knowledge Summary, reviewing practice protocols, performing a clinical audit.

Participants expressed a desire for visual aids to ease assimilation of the information, and to break up or replace large blocks of text. Other requests included starting with the learning objectives and summarising key points at the end of each section. Quizzes were a popular feature as was the use of case examples, demonstrating application of the techniques, as the material would be easier to understand and more enjoyable to read.

‘I quite like (quizzes) as a way of making sure you have learned it, you have understood it’. (11)

‘I think sometimes it really helps to go through a case, as well. And if you think of it in terms of a case, you might remember it a bit more than just going through things’. (16)

Another suggestion was for training to be available in different forms e.g., podcasts and webinars. This was particularly important for farm and equine practitioners who mentioned that time spent driving to calls was useful for catching up on CPD.

Many of the participants felt that the length of the overall resource was not as important as the ability to break it down into manageable portions.

‘Yes, it wouldn’t necessarily matter how long it was overall if it was broken up into hour or half hour chapters […]’. (17)

Ensuring time spent on the resource would count towards the RCVS CPD requirement was important to many of the participants. In addition, it was felt by several participants that ‘one-size-fits-all’ was not appropriate, given the varying EBVM skills within the profession and the different levels of interest in the subject. A proposed approach was to create a succinct resource to convey only the basic skills and ideas, with additional content provided for those who would like to take their interest further e.g., links to other resources, additional reading materials, and forums for discussion.

### Other ways to remove barriers / challenges

Some ideas were beyond the scope of this project. For example, developing a nationwide, or even international evidence resource like the Cochrane Library of Systematic Reviews available for human medicine. Participants mentioned being envious of the quantity, quality, and accessibility of human medical research.

Further recommendations revolved around ensuring that acquiring the skills to practise EBVM was a compulsory part of becoming a competent veterinary practitioner in the future. There were several suggestions including further increasing the teaching and emphasis on EBVM skills during university courses, requiring the use of EBVM through the RCVS Practice Standards Scheme, and promoting the benefits of EBVM within the veterinary community to encourage greater use and engagement.

### Developing a new online resource: ‘EBVM for Practitioners’

As a result of the focus group findings and the discussions at the workshop hosted by RCVS Knowledge, a new online resource ‘EBVM for Practitioners’ was designed. It is a slim-line resource for busy practitioners, focusing on basic training in the key skills required for EBVM, and offering advice that supports activities practitioners identified as important e.g., clinical audits and journal clubs. It includes signposting to other open-access resources and links to ‘EBVM Learning’ for those practitioners seeking to further develop their skills in EBVM. ‘EBVM for Practitioners’ is available at: www.ebvmforpractitioners.org

## Discussion

This study sought to explore practitioners’ motivations for using EBVM, and the barriers and challenges they encountered. The study findings informed the design of a new online EBVM training resource (‘EBVM for Practitioners’) to help practitioners optimise their use of evidence in clinical decision-making. Increasing the application of evidence is seen as a key way that EBVM can drive improvements in clinical care (Dean & Heneghan, 2019).

During the focus group discussions, the main reasons cited by the participants for not being able to undertake more EBVM as part of their daily practice were insufficient time, a lack of support for EBVM from colleagues or practice management, inadequate access to evidence, and limited skills in EBVM (particularly acquiring and appraising evidence). These key challenges to implementing EBVM align with those identified by several previous studies (Vandeweerd et al, 2012; Zwolsman et al. 2013; Hauser & Jackson, 2017; and Sellers et al., 2021). Pupport to overcome these challenges should be instrumental in driving an increase in EBVM in the future.

The participants were clearly conscious of the value of EBVM in general practice, but greater promotion and overall awareness of these benefits would be helpful. It has previously been shown that support of EBM / EBVM in clinical settings is associated with increased uptake (Zwolsman et al., 2013; and Sellers et al., 2021). Promoting EBVM to veterinary practitioners and the wider veterinary profession could focus on highlighting the benefits e.g., improving clinical outcomes, increasing practitioners’ confidence, building client trust, and increasing employee engagement (Hauser & Jackson, 2017).

The participants identified a need for more accessible, relevant evidence of sufficient quantity and quality, and described their EBVM activities as centring around group knowledge-sharing (e.g., journal clubs) or practice-wide uses of EBVM (e.g., clinical guidelines). It is recognised across the profession that various improvements to the veterinary evidence-base are needed (Lanyon, 2014; Dean & Heneghan, 2019; and Sellers et al., 2021), which would allow practitioners to utilise EBVM more in their day-to-day practice e.g., when deciding on treatment of an individual case. Greater use of evidence syntheses and published evidence-based clinical guidelines are ways to reduce the time burden on busy practitioners and avoid the need to undertake a full independent evaluation (appraisal) of the primary sources. There have been advances made within the profession to increase the number and accessibility of this type of evidence (as previously outlined in the Introduction), but more should be done to expand and promote these to practitioners if their use is to become widespread.

Primary or secondary sources of evidence were rarely mentioned by the participants as being used in practice, reflecting the findings of Vandeweerd et al. (2012). Access to evidence databases was limited for those working outside an academic setting, with many relying instead on the convenience of textbooks or consulting with more experienced colleagues. However, there was an awareness of the limitations of the evidence sources being used and a desire to apply higher-quality evidence more often in practice. Over two thirds of practitioners (272/403) in a recent study (Hauser & Jackson, 2017) felt that clinical practice was often based on anecdotal evidence, suggesting that there is significant potential to increase the use of evidence to inform clinical decision-making. Raising awareness of the existence of high-quality secondary sources e.g., evidence syntheses, and providing guidance on how to acquire primary research were both incorporated into ‘EBVM for Practitioners’, with the aim of improving the quality of evidence that practitioners had access to.

It was clear from the focus groups that current veterinary undergraduate curricula in the UK are increasingly providing the basic training on EBVM. However, the participants were still keen to improve their knowledge as having more skills in EBVM was associated with an increased use in practice, even where barriers and challenges were still present (e.g., lack of time, support, or access to evidence). EBVM is a vital clinical skill and requires ongoing development and consolidation (Huntley et al., 2017), which can be supported by ensuring EBVM teaching and the promotion of its value continue to be embedded within both undergraduate and postgraduate training (Arlt et al., 2012; Steele et al., 2013; Dean et al., 2017; Huntley et al., 2017; Gibbons et al., 2021; and Batt-Williams & Lumbis, 2022). The ‘EBVM for Practitioners’ resource will be an accessible way to provide a succinct summary of the key skills required for EBVM, as an initial introduction to the subject, or as a refresher course.

In the future, the participants hoped that the veterinary profession would follow the medical profession, with widespread access to free, relevant, up-to-date evidence syntheses being seen as key to transforming healthcare (Smith & Chalmers, 2001). There are many ways in which the wider veterinary profession is rising to the challenge of promoting and supporting the use of EBVM; the production of resources and provision of practical support by several key organisations such as RCVS Knowledge and the Centre for Evidence-based Veterinary Medicine (CEVM), the open-access online training tutorial ‘EBVM Learning’, and the inclusion of EBVM in the RCVS Day One Competences and RCVS Practice Standards Scheme (2022) within the UK.

### Limitations

The study was undertaken in 2020, and as such was influenced by the global Covid-19 pandemic. Practitioners were extremely busy, making recruitment more difficult than originally anticipated, which reduced the sample size for the focus groups. Due to limitations of the sampling method and sample size, it was not possible to ensure that the focus group participants reflected the broader demographic of veterinary practitioners in terms of years of experience and type of work undertaken. The selection of practitioners was limited to the network of the research team, so creating a potential bias towards those who had more experience / knowledge of EBVM. The resultant sample was biased towards those who had graduated more recently (63.6% graduated in the past 10 years), and those working as veterinary surgeons (only 9.1% of participants were veterinary nurses).

If further research was to be conducted it would be valuable to attempt to recruit a focus group population that more closely represented the veterinary profession as a whole, to ensure that the views of all members of the profession and their needs are heard and considered. However, it is noted that generalisability is not a specified aim within qualitative research; rather enough details are provided so that readers can understand what aspects of the findings might be transferable to their own context.

All focus group participants were currently working within clinical practice in the UK, which may limit the generalisability of the findings to an international context.

### Future work

There are various opportunities for future research, in particular to investigate the impact of any intervention that aims to promote EBVM. It would be interesting to evaluate ‘EBVM for Practitioners’ by measuring the use of EBVM prior to and after accessing the resource, and by gathering feedback on additional support that practitioners would find useful once they have tried to put EBVM into practice.

### Conclusion

In conclusion, there are many challenges and barriers to the use of EBVM in general practice; participants identified time, support from colleagues and management, and accessing and appraising evidence as being the most important. However, the potential value of EBVM was clear, and that it could provide benefits to practitioners, practices, and the whole veterinary profession. Practitioners are keen for more of their clinical decisions to be supported by robust evidence, to provide better healthcare, and to feel more confident in their clinical work.

There are several initiatives within the veterinary profession that are aiming to place EBVM as a central part of clinical practice, but more can be done to expand and improve the quality of the evidence base on which EBVM relies, and to ensure practitioners have the skills, access, and motivation to utilise it. This study has been used to produce ‘EBVM for Practitioners’, to attempt to reduce some of the barriers and challenges faced by practitioners and support them in increasing their use of EBVM.

### Supplementary materials

Supplementary Material S1 – Focus Group Topics and Questions

### Acknowledgements

We would like to thank the practitioners who participated in the focus groups for giving their valuable time, and the project team who attended the workshop and provided contributions to the online resource development. We would like to give particular thanks to Clare Boulton, Rachel Dean, Joanne Ireland, and Heather Moberly for their contributions to reviewing and editing ‘EBVM for Practitioners’, and to RCVS Knowledge for supporting and funding the project. 

### Author contributions

**Laura Haddock:** Conceptualisation, Methodology, Validation, Formal analysis, Investigation, Resources, Data Curation, Writing – Original Draft, Writing – Review & Editing, Visualisation, Project administration, Funding acquisition. **Sarah Baillie:** Conceptualisation, Methodology, Validation, Formal analysis, Data curation, Writing – Original draft, Writing – Review & Editing, Visualisation, Supervision, Project administration, Funding acquisition. **Ellie Sellers:** Conceptualisation, Writing – Original draft, Writing - Review & Editing. **Sheena Warman:** Conceptualisation, Methodology, Validation, Formal analysis, Data curation, Writing – Review & Editing, Supervision.

### ORCID

Laura Haddock: https://orcid.org/0000-0002-7849-6953

Sarah Baillie: https://orcid.org/0000-0002-8665-8369

Ellie Sellers: https://orcid.org/0000-0002-3809-9777

Sheena Warman: https://orcid.org/0000-0003-0829-2039

### Conflict of Interest

The authors declare no conflicts of interest.
